# Surgical treatment of primary intracranial and extracranial communicating leiomyosarcoma: a case report

**DOI:** 10.3389/fonc.2025.1510221

**Published:** 2025-03-17

**Authors:** Kuairong Pu, Tianhong Wang, Zhe Li, Xiwen Lin, Jun Wu, Dongchuan Shao, Nan Zhao

**Affiliations:** ^1^ Department of Neurosurgery, First People’s Hospital of Kunming, Kunming, China; ^2^ Department of Neurosurgery, Department of Jinning District People’s Hospital, Kunming, China

**Keywords:** primary intracranial tumor, leiomyosarcoma, clinical features, surgical removal, case report

## Abstract

Primary intracranial-extracranial communicating leiomyosarcomas, capable of invading both intracranial and extracranial regions and involving complex anatomical structures, are exceedingly rare neoplasms. We present the case of a 37-year-old male initially presented with a subcutaneous mass on the left frontal vertex. Post-surgical intervention, a recurrent lump emerged on the left frontotemporal vertex. Symptoms, computed tomography (CT), and magnetic resonance imaging (MRI) revealed a mass on the left frontal vertex accompanied by an irregular abnormal lesion. Leiomyosarcoma diagnosis was confirmed on both occasions. The patient underwent leiomyosarcoma excision under general anesthesia. Recurrence was noted 2 years and 4 months post-surgery, necessitating an expanded excision. After 2 years of follow-up, no significant complications were observed, and the patient’s condition remains stable. Primary extracranial communicating leiomyosarcoma is exceptionally rare, with surgery as the primary treatment modality. The decision to excise the lesion should consider the patient’s age, tumor location, pathological features, and presence of distant metastases.

## Introduction

Primary intracranial leiomyosarcomas, rare smooth muscle sarcomas within the cranial cavity, account for merely 0.1% to 0.2% of all intracranial tumors (1). They can affect individuals of any age and gender, presenting various symptoms based on tumor location, such as memory loss, hemiparesis, and seizures (2). Imaging studies typically show an irregular mass with heterogeneous enhancement on MRI, originating from the dura mater or cerebrum (3).

Histologically, these tumors are similar to soft tissue leiomyosarcomas, with interwoven spindle-shaped cells, and occasional fence-like or perivascular epithelioid cell tumor-like arrangement (4). The cells are densely packed, sometimes showing fibrosis and myxoid alterations. Larger tumors often exhibit hyalinization and coagulative necrosis (5). The precise etiology remains elusive, but associations with immunosuppression and viral infections are reported (6). This neoplasm is generally aggressive with a poor prognosis.

Treatment strategies typically depend on the patient’s age, tumor location, pathological characteristics, and presence of distant metastases, including surgical resection, radiotherapy, chemotherapy, and biological therapy, with surgical resection being primary (7). These may include surgical resection, radiotherapy, chemotherapy, and biological therapy, with surgical resection serving as the primary treatment modality (8). With over 30 surgical treatment reports currently available, this case report discusses the clinical characteristics and treatment experience of a rare primary intracranial-extracranial communicating leiomyosarcoma. The patient underwent surgical resection in 2015, experienced a recurrence 2 years and 4 months later, treated with an expanded resection. As of 2023, no signs of recurrence were noted, underscoring the efficacy of surgical intervention as the main treatment approach.

## Case report

A 37-year-old married male patient was admitted to our hospital on May 10, 2015, with a gradually enlarging subcutaneous mass on the left frontal vertex, present for 1 month. Examination revealed a pliable, non-tender mass measuring approximately 4 cm x 5 cm with a clear texture and well-defined borders, elevated about 0.7 cm above the normal skin margin.

CT indicated local lytic changes in the left frontal bone, with spindle-shaped hypo-to isodense shadows seen on the inner and outer aspects of the skull. The partial brain tissue was slightly compressed and shifted inward, with uneven density within and a CT value of about 38 HU. Uneven enhancement was observed at the edges.

**Figure 1:**
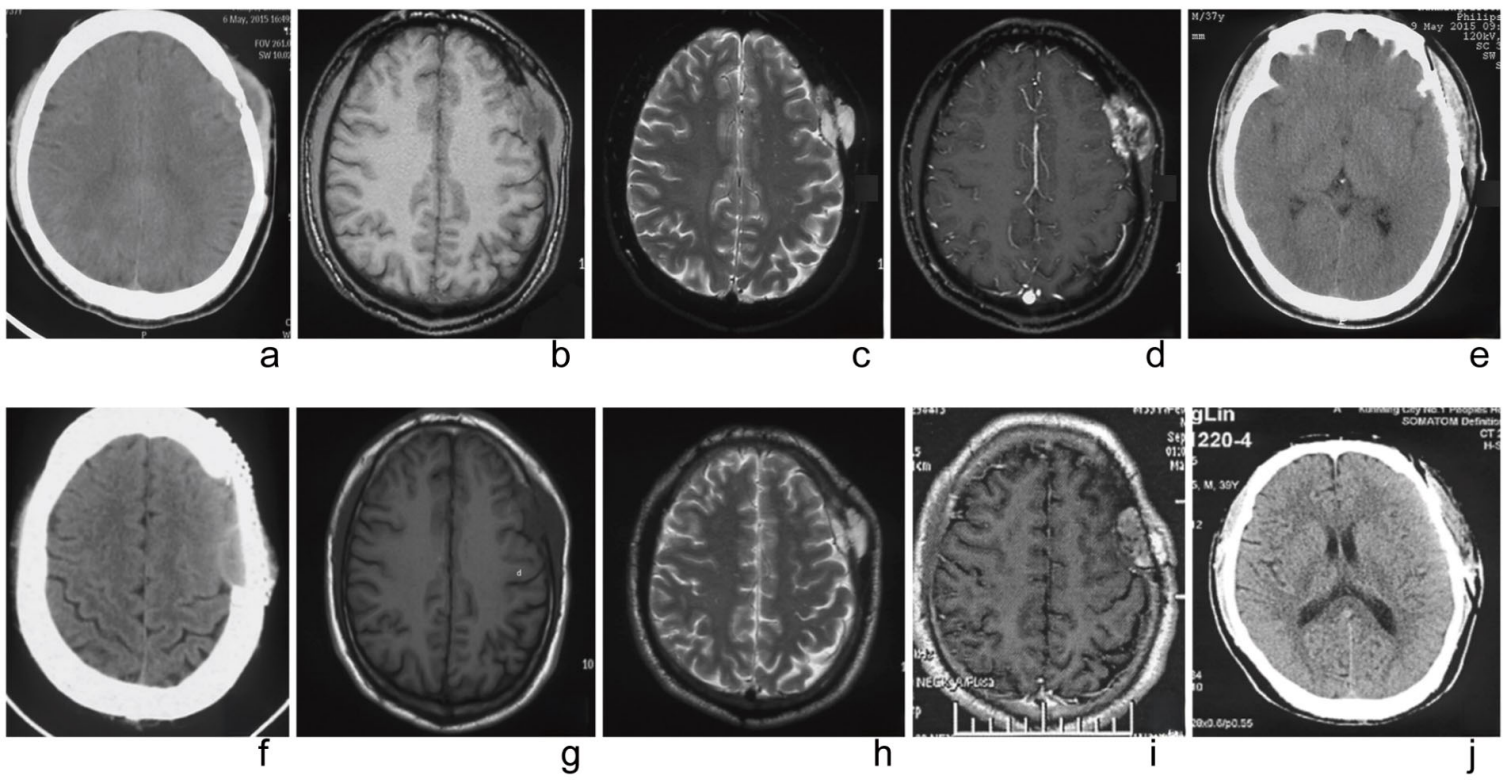
The imaging findings before and after the first surgery. **(a)** Preoperative CT showed local cranial bone with moth-eaten changes and a spindle-shaped to hypodense shadow on the lateral side of the skull, with uneven density inside. **(b)** Preoperative MRI (T1WI) displays an uneven signal density. **(c)** Preoperative MRI (T2WI) appears as an isointense to hyperintense signal. **(d)** Preoperative MRI enhancement shows ring-like enhancement. **(e)** A one-week postoperative follow-up cranial CT indicates complete tumor resection. The imaging findings before and after the second surgery. **(f)** Preoperative CT reveals a low-density lesion with a wide base and clear boundary. **(g)** Preoperative MRI (T1WI) shows the soft tissue as a lower-density shadow. **(h)** Preoperative MRI (T2WI) presents as an isointense to hyperintense signal. **(i)** Preoperative MRI enhancement demonstrates uneven enhancement. **(j)** A one-week postoperative follow-up cranial CT indicates complete tumor resection.

MRI revealed a spindle-shaped lesion with equal T1 and long T2 signals in the left frontal vertex bone, measuring approximately 2.2 cm x 3.5 cm x 4.0 cm, centered on the skull bone with associated bone absorption and thinning. The lesion displayed uneven low signals indicating bone destruction, and abnormal uneven enhancement was observed after contrast administration, with significant enhancement on delayed scanning. The adjacent brain parenchyma was slightly compressed and shifted inward (**Figures 1a–e**).

**Figure 2:**
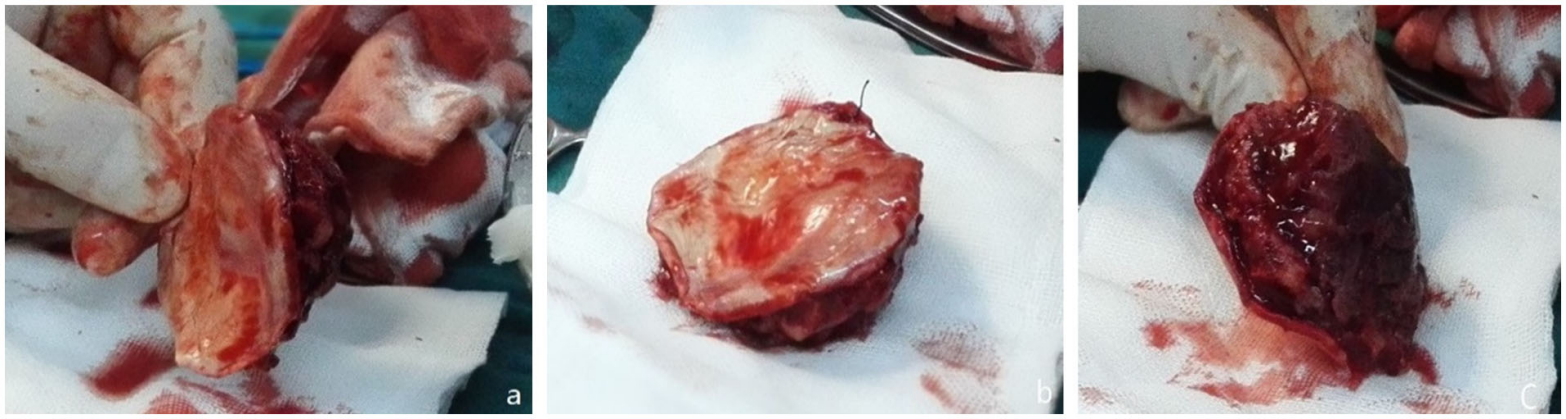
During the first surgery. **(a)** Leiomyosarcoma tissue excised **(b)** Affected dural surface. **(c)** The inner surface of the skull.

During surgery, a mass in the left frontal area was observed to have a fish-flesh appearance, firm texture, and no significant adhesion to the scalp. An intraoperative frozen section examination was performed. After fully exposing the extracranial tumor, the skull was drilled, and the bone was bitten along the tumor. A circular incision was made in the dura mater. Due to the lack of adhesion between the tumor and the brain, the entire tumor was removed (**Figure 2**). Following the intraoperative frozen section examination, which suggested a malignant tumor, the scope was expanded, and approximately 2 cm of surrounding tissue was removed. A range of about 8 cm x 8 cm of normal skull and dura mater was excised, and a phase-one artificial dura mater and titanium mesh repair were performed.

The diagnosis of leiomyosarcoma was confirmed by the presence of spindle-shaped and round cells irregularly arranged within the tumor, with abnormally enlarged, deeply stained nuclei showing significant atypia. A few vacuolated cells were observed, with abundant acidophilic cytoplasm and visible mitosis, forming diffuse plaques and some interstitial mucoid degeneration.

**Figure 3:**
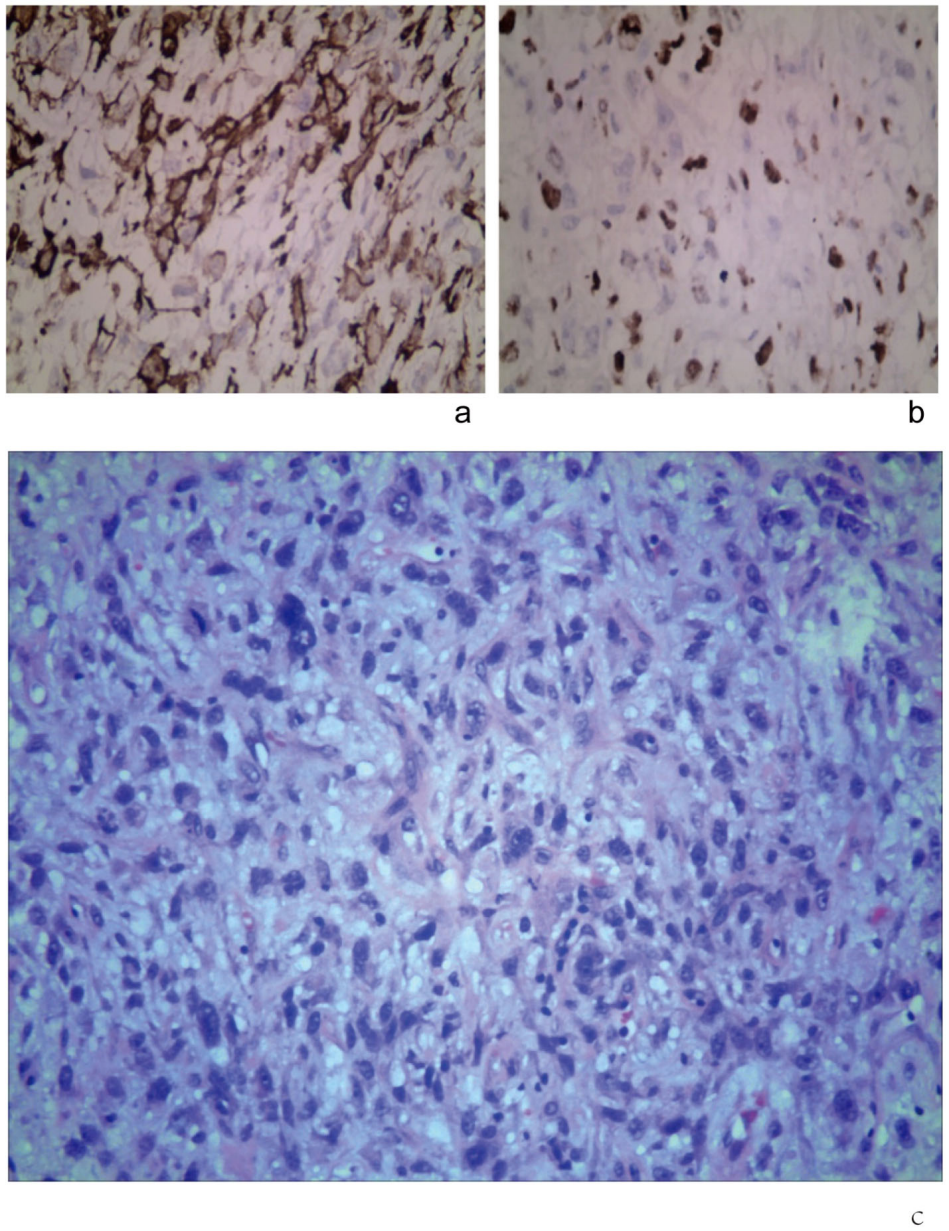
Immunohistochemical staining (original magnification, × 400). **(a)** SMA (+). **(b)** Ki-67 (+). **(c)** Tumor cells were under the microscope after the second surgery, with deeply stained nuclei, significant pleomorphism, and evident mitotic figures (HE, ×400).

Immunohistochemistry results indicated positive silver staining, B-cell lymphoma-2 (Bcl-2), CD34, FLI-1, FN, and SMA, with scattered CD68, and Ki-67 at 30%. The specimen’s margins contained normal tissue, displaying tumor cells arranged in irregular spindle shapes with enlarged nuclei and some showing a vacuolar appearance (**Figures 3a, b**).

The patient was followed up 9 days after discharge. Since the tumor was completely excised and was not sensitive to radiotherapy and chemotherapy, these treatments were not administered. Routine follow-ups over 2 years and 3 months until August 2017 showed no recurrence, and PET-CT scans during this period did not reveal any recurrence or distant metastasis. However, at 2 years and 4 months postoperatively, a subcutaneous mass appeared on the left frontotemporal vertex, which was considered a recurrence of the leiomyosarcoma.

The patient was readmitted on September 27, 2017, due to a lump on the left frontotemporal vertex, present for 20 days. Examination revealed an original surgical scar on the left frontotemporal vertex and a subcutaneous mass approximately 3 cm in diameter, with a pliable texture, clear boundaries, no tenderness, redness, or skin ulceration, and elevated about 0.5 cm above the normal skin margin. No vascular murmur was detected within the mass. Enlarged lymph nodes were palpable on both sides of the neck, with the largest measuring about 3 cm in diameter.

CT scans indicated the absence of bone substance in the left frontotemporal vertex, revealing a broad-based segment of low-density shadow with clear boundaries, measuring approximately 3.2 cm × 1.3 cm, and a CT value of about 39 HU. MRI demonstrated a mass and post-surgical changes in the left frontal vertex, showing an irregular abnormal lesion in the inner table of the left parietal bone. This lesion presented as equal T1 and slightly long T2 signals, measuring about 3.5 cm × 1.6 cm × 3.5 cm, with uneven enhancement after contrast administration and significant mass effect (**Figures 1f–j**).

During the surgery, a mass of approximately 3 cm × 4 cm was observed attached to the three-dimensional titanium mesh on the left frontal vertex, with no clear demarcation from the surrounding tissue. The mass was removed in sections. After the titanium mesh was removed, a 4 cm mass was noted on the left parietal vertex. The excised mass had a fish-flesh appearance with clear demarcation. The artificial dura mater, tumor tissue, and affected skull in that area were excised. The tumor originated from the dura mater at the vertex. An additional 3 cm of skull, dura mater, periosteum, and subcutaneous tissue towards the vertex were removed. Towards the frontotemporal area, 4 cm from the tumor, proliferative tissue, artificial dura mater, brain tissue, and skull were excised, along with a skin flap measuring 2 cm × 5 cm at the tumor site. The surgical procedure involved a phase-one repair with artificial dura mater and titanium mesh.

The diagnosis confirmed leiomyosarcoma (WHO Grade III). The tumor contained spindle-shaped and round cells irregularly arranged, with deeply stained and enlarged nuclei. Hematoxylin and Eosin (HE) staining revealed significant pleomorphism, a few vacuolated cells, and conspicuous mitotic figures diffusely distributed in sheets, with some interstitial mucoid degeneration (**Figure 3c**). Immunohistochemistry studies showed positive results for silver immersion staining and SMA. No tumors were found in the surrounding tissues, and cytology from needle aspiration biopsy of the neck lymph nodes showed no tumor cells.

The patient was discharged 10 days after surgery. Given the recurrence of leiomyosarcoma without metastasis, no radiotherapy or chemotherapy was administered before or after the surgery. The patient has been regularly returning for check-ups, and as of June 1, 2020, has been followed up for over 2 years and 8 months, with no significant complications reported. Follow-up continues.

## Discussion

Leiomyosarcoma is a rare malignant tumor originating from mesodermal tissues, commonly found in the uterus, gastrointestinal tract, and retroperitoneum. Head and neck leiomyosarcomas account for 1% to 4% of all leiomyosarcomas (1, 9). Primary intracranial-extracranial communicating leiomyosarcomas are exceedingly rare, often originating from the meningeal interstitium, and are unrelated to meningeal epithelial cells (10). These tumors can occur at any age and show no significant gender difference, typically appearing in individuals with compromised immune function (11), especially in HIV-positive patients carrying the EB virus (12). Intracranial sarcomas are mostly found supratentorial and very rarely in the cerebellum or spinal cord, with symptoms varying depending on the tumor location. Extracranial leiomyosarcomas often present as subcutaneous masses, while intracranial leiomyosarcomas commonly cause symptoms such as headaches, vomiting, and secondary epilepsy. In this case, the patient presented with a subcutaneous mass without related intracranial symptoms.

Several case reports and studies have documented similar cases of primary intracranial-extracranial communicating leiomyosarcomas, though their rarity makes comprehensive comparison challenging. Li et al. (2019) reported a case of primary intracranial leiomyosarcoma in an immunocompetent patient, emphasizing the importance of radiological features in diagnosis and surgical planning. Their case shared similar imaging characteristics with ours, particularly the pattern of bone invasion and enhancement on MRI (6). The 5-year survival achieved in our case is particularly noteworthy, as previous studies have reported varying survival rates. For instance, Zhang et al. (2020) reviewed 15 cases of primary intracranial leiomyosarcomas, finding a median survival time of 23 months, making our patient’s outcome relatively favorable in comparison (13).

Clinical diagnosis relies on the imaging characteristics of the tumor and pathological immunohistochemistry results. On CT, leiomyosarcomas typically appear as medium-sized soft tissue masses with clear boundaries, although some parts may exhibit infiltrative growth with blurred edges. MRI usually shows uneven signals with some cystic changes and necrosis, and ring-like enhancement at the edges after contrast (12). In this case, the sarcoma was clearly demarcated from the normal brain tissue, with only mild edema at the edges, uneven internal signals, and obvious uneven enhancement after MRI contrast, with non-enhanced flaky areas visible inside. Pathological examination revealed that intracranial-extracranial communicating leiomyosarcomas, similar to soft tissue leiomyosarcomas elsewhere, are primarily arranged in irregular spindle cell bundles. Microscopically, they exhibit significant pleomorphism, elongated and variable nuclei with deep staining, easily visible mitotic figures, acidophilic or pale cytoplasm, and a common vacuolated appearance. Immunohistochemistry typically shows strong diffuse positivity for SMA, calponin, and desmin, with approximately 30% Ki-67 positivity (14).

Primary intracranial-extracranial communicating leiomyosarcomas need to be differentiated from related intracranial and extracranial lesions, such as leiomyosarcoma (LMS), brain metastasis (BM), hemangiopericytoma (HPC), epithelioid glioblastoma (EGB), osteosarcoma (OS), gliosarcoma (GS), and fibrosarcoma (FS) (**Supplementary Table 1**) (15, 16).

The primary treatment for leiomyosarcoma remains surgical excision, removing all visible tumor tissue and ensuring negative pathological margins intraoperatively, followed by expanding the excision to include more than 2 cm of normal tissue (17, 18). Late-stage tumors are highly malignant, with a short disease-free survival period, typically between 6 to 24 months (19). Complete removal via surgery is challenging, so radiotherapy may be added before or after surgery (20). Studies have shown that bevacizumab can effectively treat certain soft tissue sarcomas (21), but insufficient clinical data supports its use for primary intracranial-extracranial communicating leiomyosarcomas.

In this case, the patient had bone destruction, and the first expanded excision surgery was effective. After recurrence, two additional surgeries further expanded the excision range by 3 to 4 cm. As of June 1, 2020, follow-ups showed no recurrence, and the patient has survived for 5 years since the first surgery, indicating that surgical excision remains the main treatment method for this tumor. Currently, there is no uniform standard for the range of surgical excision, and it is recommended that even if the intraoperative rapid pathological examination shows negative margins, the excision range should still be expanded by more than 2 cm.

This case report is significant for documenting a rare primary intracranial-extracranial communicating leiomyosarcoma with detailed imaging, surgical, and follow-up data, with our patient’s exceptional 5-year survival following aggressive surgical management exceeding the typically reported median survival of 23 months, while also demonstrating successful management of tumor recurrence through expanded surgical resection. Our experience has important clinical implications, suggesting that aggressive surgical resection with margins exceeding 2 cm may improve outcomes even in recurrent cases, while the successful outcome without adjuvant therapy indicates surgery alone may suffice in selected cases with complete resection; furthermore, our case emphasizes the necessity of long-term surveillance, as recurrence occurred at 2 years and 4 months post-initial surgery. While the study’s main limitation is its nature as a single case report involving an immunocompetent patient, which limits generalizability, its strengths include comprehensive documentation of imaging, pathological findings, and surgical approach, along with extended follow-up data showing successful outcome—most notably, the patient’s 5-year survival provides evidence that aggressive surgical management can achieve favorable outcomes in these rare tumors.

## Data Availability

The original contributions presented in the study are included in the article/**Supplementary Material**. Further inquiries can be directed to the corresponding author.
