# Supplementary figures and images for: Surgical treatment of primary intracranial and extracranial communicating leiomyosarcoma: a case report
Source: Front Oncol. 2025 Mar 17;15:1510221. doi: 10.3389/fonc.2025.1510221 (PMC11955503; doi:10.3389/fonc.2025.1510221)

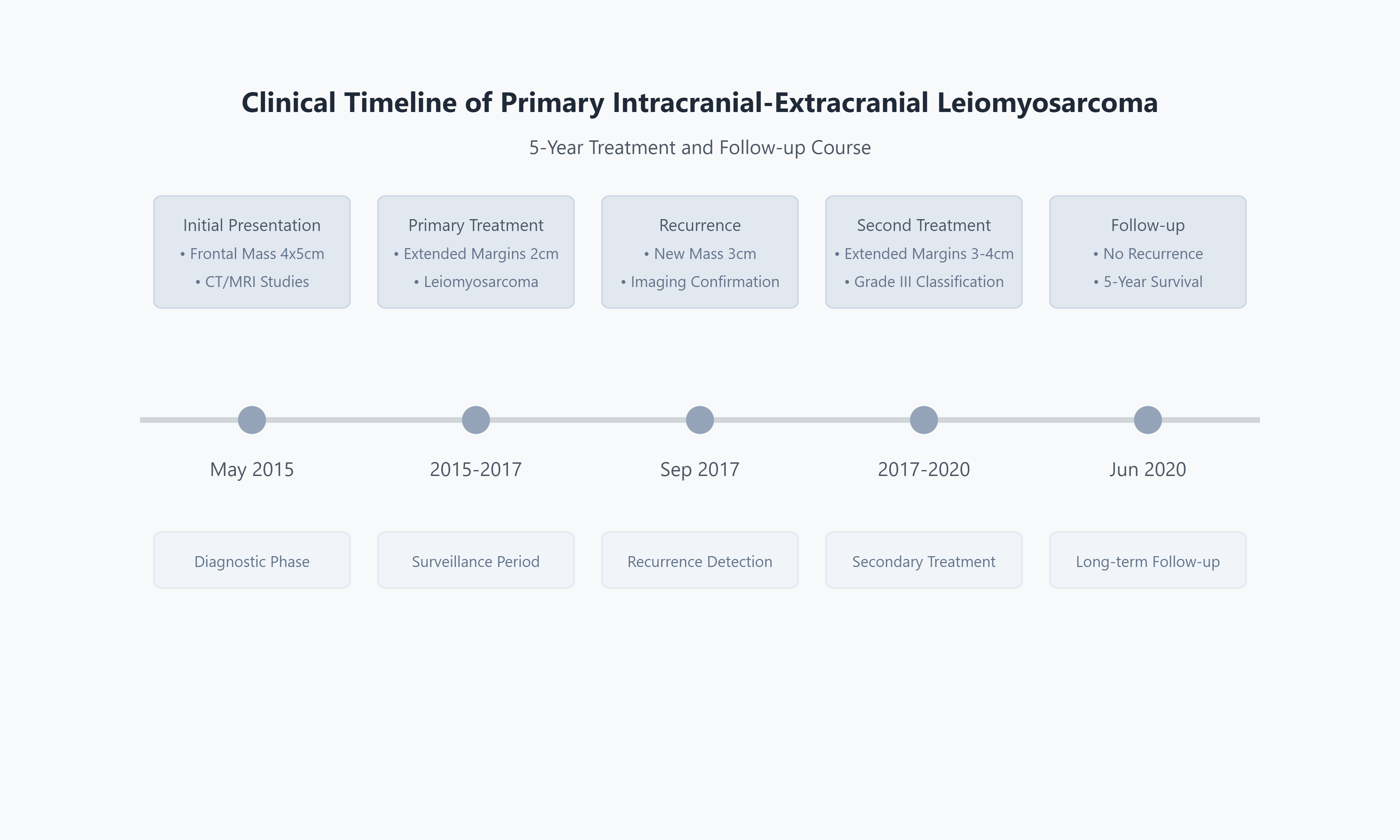

Supplement: Supplementary file 1 [file Image1.png]
