# Supplementary material for: Surgical treatment of primary intracranial and extracranial communicating leiomyosarcoma: a case report
Source: Front Oncol. 2025 Mar 17;15:1510221. doi: 10.3389/fonc.2025.1510221 (PMC11955503; doi:10.3389/fonc.2025.1510221)
Supplement: Supplementary file 2 [file Table1.docx]

Table 1 Differential diagnosis associated with primary intracranial and extracranial communicating smooth muscle sarcoma

| Pathological characteristics | LMS | BM | HPC | EGB | OS | GS | FS |
| --- | --- | --- | --- | --- | --- | --- | --- |
| Ages | 40~60 | 40~50 | 30~45 | 5~10 | ＜20 | 40~60 | 20~40 |
| Sexs | Female | Female | Male | Male | Female | Male | Male |
| Times | Short | Long | Short | Long | Short | Short | Short |
| Cranial Changes | Erosion damage | deformity | Entomophagous bone destruction | Limited osteolytic destruction | Osteophyte, osteolytic destruction | Erosion of the bone periosteum | Proliferation,malformation |
| CT | Large lobulated mass, predominantly solid, with cystic and necrotic areas, rare calcification, heterogeneous enhancement on enhancement, frequent collateral circulation | Slightly dense round-like shape with clear surrounding border, the tumor mostly shows uniform moderate enhancement after enhancement, the occupying sign is obvious, and the difference of peritumor edema is big | Irregular lobulated, high, equal and low density on plain scanning, unclear border, common cystic degeneration and necrosis, peritumor edema is not obvious, homogeneous and significant enhancement after enhancement | Visible bone destruction, usually involving both the inner and outer cranial plates, with variable and irregular morphology and well-defined osteolytic bone destruction | Irregularly shaped soft tissue mass, often accompanied by bone destruction, with the presence of tumor bone in the mass | Mixed density shadows, large lesions, mostly involving multiple lobes, cystic necrosis can be seen in the foci, peritumor is often accompanied by edema, with clear boundaries after enhancement and irregular moderate enhancement. | Circular or lobulated mass with uneven density, blurred border, may be accompanied by hemorrhagic necrotic cystic changes, mild to moderate peritumoral edema, uneven enhancement after enhancement |
| MRI | Low signal on T1-weighted images, high signal on T2-weighted images, and significant enhancement on enhancement | Equal or slightly longer T1, long or slightly longer T2 signals, markedly homogeneous enhancement after enhancement, visible “meningeal tail sign”. | T1WI and T2WI showed equal and slightly low signals, cystic necrosis was seen in the lesion, accompanied by empty blood vessels, the tumor boundary was clear, and slight edema was seen in the periphery of the lesion. | The lesion was isosignal on T1WI, slightly high signal on T2WI with some punctate slightly low signal, and moderate or high signal on T2Flair, with inhomogeneous enhancement. | T1WI tends to be isosignal, T2WI tends to be isohigh signal, often clearly identified by typical Codman's triangle | T1WI showed irregular long T1 slightly low signal and T2WI showed mixed high signal with inhomogeneous parenchymal enhancement [ | Low or equal signal in T1WI, mixed signal in T2WI, and obvious patchy enhancement after enhancement |

Table 2 abbreviations

| Word | Abbreviation |
| --- | --- |
| Magnetic Resonance Imagingm | MRI |
| Computed Tomography | CT |
| T_1_-weighted Imaging | T_1_WI |
| T_2_-Weighted Imaging | T_2_WI |
| B-cell lymphoma-2 | Bcl-2 |
| Smooth Muscle Actin | SMA |
| Haematoxylin and eosin | HE |
| Human Immunodeficiency Virus | HIV |
| Epstein-Barr | EB |
| Leiomyosarcoma | LMS |
| Benign Meningioma | BM |
| Hemangiopericytoma | HPC |
| Eosinophilic Granuloma of Bone | EGB |
| Skullosteoma | OS |
| Gliosarcoma | GS |
| Fibrosarcoma | FS |
